# Immune checkpoint inhibitor resistance in soft tissue sarcoma

**DOI:** 10.20517/cdr.2021.127

**Published:** 2022-04-06

**Authors:** Vanessa Eulo, Brian A. Van Tine

**Affiliations:** ^1^Division of Medical Oncology, University of Alabama at Birmingham, Birmingham, AL 35233, USA.; ^2^Division of Medical Oncology, Washington University in St. Louis, St. Louis, MO 63110, USA.; ^3^Division of Pediatric Hematology/Oncology, Washington University in St. Louis, St. Louis, MO 63110, USA.; ^4^Siteman Cancer Center, St. Louis, MO 63110, USA.

**Keywords:** Sarcoma, soft tissue sarcoma, checkpoint inhibitor, immunotherapy, resistance

## Abstract

The emergence of immunotherapy as a cancer therapy has dramatically changed the treatment paradigm of systemic cancer therapy. There have been several trials evaluating immune checkpoint blockade (ICI) in soft tissue sarcoma. While there is generally a limited response in sarcoma, a subset of patients has durable responses to immunotherapy. This is attributable to a variety of factors including histologic subtype, tumor-infiltrating lymphocytes, and the tumor microenvironment among others. There is ongoing translational and clinical research evaluating ICI resistance in sarcoma and identifying therapeutic strategies to overcome this resistance. Herein, we provide a review of the current data, proposed mechanisms of resistance, and potential approaches to overcome this resistance.

## INTRODUCTION

Soft tissue sarcomas (STS) are a heterogenous group of cancers of mesenchymal origin^[1]^. They represent 1% of cancer diagnoses in the United States, with 13,130 diagnosed in 2020^[2]^. The prognosis of soft tissue sarcoma is poor, with up to 50% of patients with localized disease developing metastases and a median survival of 12-24 months in the advanced setting^[3,4]^. There are more than 50 histologic subtypes of soft tissue sarcoma, each with a distinct clinical profile, prognosis, and response to treatment.

Sarcomas are generally divided into two categories based on genetics, simple and complex. Many simple subtypes are translocation driven and have limited neoantigens, such as synovial sarcoma (SS) and myxoid/round cell liposarcoma^[5]^. Complex sarcomas have more complex genetics, with potentially mutated protein targets for T-cells^[5]^. The most common of these include Undifferentiated Pleomorphic Sarcoma (UPS) and Leiomyosarcoma (LMS), and they have been found to have higher gene expression levels related to antigen presentation^[5,6]^.

First-line therapy consists of anthracycline-based cytotoxic chemotherapy, and Doxorubicin remains the most active single agent with a response rate of up to 20-25%^[7-11]^. There have been attempts to improve this treatment approach with dose intensification and combination therapies, but these have had a limited impact on overall survival^[4,8]^. With the emergence and success of immunotherapy in other cancer subtypes, there has been interest in using this modality to treat soft tissue sarcomas^[6]^. While there are several modalities of immunotherapy, including T-cell transfer therapy, monoclonal antibodies, cancer vaccines, immune system modulatory and ICI, our review will focus on ICI^[12]^. This has led to multiple clinical trials of ICI and ICI combination therapy in advanced soft tissue sarcoma [Table 1].

**Table 1:** Studies of checkpoint inhibition in sarcoma

**Authors**	**Year**	**Study type**	**Treatment**	***N***	**ORR **	**mOS **	**PFS **
Tawbi *et al.*^[13]^	2017	Phase II	Pembrolizumab	*N* = 80	18% (7/40 STS)	12.25m (8.50-18.25)	4.5m (2-5.25)
D’Angelo *et al.*^[14] ^	2018	Phase II	Nivolumab	*N* = 38	5% (2/38)	10.7m (5.5-15.4)	1.7m (1.4-4.3)
D’Angelo *et al.*^[14] ^	2018	Phase II	Nivolumab plus Ipilimumab	*N *= 38	16% (6/38)	14.3m (9.6-NR)	4.1m (2.6-4.7)
Wilky *et al.*^[15]^	2019	Phase II	Axitinib plus Pembrolizumab	*N *= 33	25% (8/32)	18.7m (12-NR)	4.7m (2-9.4)
Martin-Broto * et al.*^[16] ^	2020	Phase Ib/II	Sunitinib plus Nivolumab	*N *= 68	21% (12/58)	24m	5.6m (3.0-8.1)
Pollack * et al.*^[17]^	2020	Phase I/II	Pembrolizumab plus Doxorubicin	*N *= 37	19% (7/37)	27.6m (18.7-NR)	8.1m (7.6-10.8)
Kelly *et al.*^[18] ^	2020	Phase II	Talimogene laherpavec plus pembrolizumab	*N *= 20	35% (7/20)	18.68m (12.25-NR)	4.28m (3.15-NR)
Gordon *et al.*^[19] ^	2020	Phase II	Ipilimumab, Nivolumab, Trabectedin	*N *= 41	19.5% (8/41)	> 12.5m	> 6.0m

An early phase II study evaluated the CTLA-4 inhibitor, ipilimumab, in advanced synovial sarcoma given their high expression of cancer testes antigen NY-ESO-1. Patients were treated with ipilimumab 3 mg/kg intravenously every 3 weeks for three cycles and then restaged. Six patients were enrolled and received 1-3 cycles of ipilimumab. All patients showed clinical or radiological evidence of disease progression after no more than three cycles of therapy. This study was stopped due to slow accrual and lack of activity^[20]^. 

SARC028 was a multicenter, two-cohort, single-arm, open-label phase II study evaluating the anti-PD-1 antibody, pembrolizumab, in 40 patients with advanced soft tissue sarcoma^[13]^. Patients received 200mg pembrolizumab IV every 3 weeks until progression or unacceptable toxicity. Histologies enrolled included UPS, dedifferentiated liposarcoma (DDLPS), SS, LMS. 18% (7/40, 95%CI: 7-33) of patients with soft tissue sarcoma achieved an objective response. This is clinically meaningful, although the prespecified objective response rate to meet their endpoint was 8/40 responses. The majority of these responses were in patients with UPS or DDLPS, and one patient with UPS achieved a confirmed complete response. The 12-week PFS in the soft tissue sarcoma cohort was 55%. The median duration of response was 49 weeks and overall survival (OS) was not reached in patients with UPS. In this study, adequate tumor biopsies were obtained from 81% of patients during treatment and were analyzed for pre-treatment PD-L1. PD-L1 was positive at the 1% threshold in only three samples, all of which were from patients with UPS. Two of the three were evaluable for response, and one had a complete response and the other had a partial response^[13]^. 

There was also a single center, phase II study evaluating nivolumab, an anti-PD-1 antibody, in twelve patients with advanced uterine leiomyosarcoma^[21]^. Patients received 3 mg/kg every 2 weeks intravenously of nivolumab until progression or unacceptable toxicity. There were no responses noted in this cohort, and the second stage was not opened due to lack of benefit^[21]^.

The Alliance A091401 trial was an open-label, non-comparative, randomized, phase II study of nivolumab with or without ipilimumab, an anti-CTLA-4 antibody, in metastatic or locally advanced sarcoma^[14]^. Patients received intravenous nivolumab 3 mg/kg every 2 weeks or nivolumab 3 mg/kg plus ipilimumab 1mg/kg every three weeks for four doses followed by nivolumab 3 mg/kg every two weeks for up to two years. The most common sarcoma subtypes across both groups were leiomyosarcoma (34%), liposarcoma (6%), spindle cell sarcoma (13%), undifferentiated pleomorphic sarcoma (13%) and bone sarcoma (11%). In the nivolumab group (38 patients), the response rate was 5% (2/38, 95%CI: 1-16) and thus, did not meet its primary endpoint of objective response rate. The combination group (42 patients) had an overall response rate of 16% (6/38, 95%CI: 7-30) with a median PFS of 4.1 mo and OS 14.3 mo^[14]^. 

The results of clinical trials using checkpoint inhibition in soft tissue sarcoma have been varied, and there is an ongoing study into which sarcoma subtypes are more responsive to checkpoint inhibitors and if there are predictive biomarkers that can be used. This remains a challenge given the heterogeneity of different sarcoma subtypes and the rarity of each disease. Unlike other cancer types, biomarkers such as tumor mutational burden and PD-L1 expression have failed to identify good responders in soft tissue sarcoma. Although a small proportion of patients do respond to checkpoint inhibitors, the majority do not, likely due to primary resistance to checkpoint inhibition. In those who do respond, most will progress, which is likely due to acquired resistance. 

Herein, we will highlight here mechanisms of resistance, research evaluating predictive biomarkers in STS and current approaches to overcome resistance to checkpoint inhibition. 

## RESISTANCE MECHANISMS

Unlike other more immunogenic cancer types such as melanoma, only a minority of patients with soft tissue sarcoma develop durable clinical responses to ICI (primary resistance) and most of those who do respond will eventually progress (acquired resistance). In order for the immune system to respond effectively, cancer cell-specific antigens that are recognizable by antigen-presenting cells are required, T cells must be primed and activated with the ability to infiltrate tumors, and cancer cells must recognize and destroy cancer cells^[22]^. There also must be a balance of stimulatory and suppressive signaling that favors continued cytotoxicity by T-cells within the tumor microenvironment (TME)^[15,22]^. 

Tumors are generally thought of as immunogenic (or hot) or non-immunogenic (or cold). Sarcomas are generally characterized as non-immunogenic, cold tumors with limited immune cell infiltrate, low tumor mutational burden (TMB) and low PD-L1 expression, which is thought to contribute to their primary resistance to ICI^[23,24]^. This is by tumor intrinsic or extrinsic mechanisms. Tumor intrinsic mechanisms include lack of antigenic proteins, lack of antigen presentation, genetic T-cell exclusion or insensibility to T-cells^[25,26]^. Oncogenic signaling pathways also contribute to tumor intrinsic resistance to ICI^[26]^. Tumor extrinsic mechanisms include the absence of T cells, inhibitory immune checkpoints and immunosuppressive cells including tumor-associated macrophages (TAMs) and T-regulatory cells (Tregs)^[25]^. Upregulation of Tregs can induce immunologic tolerance and an increase in M2 TAMs suppress the TME and correlate with progression^[12]^. Within STS, there is wide heterogeneity, with response rates that are variable among histologies. Prior studies have shown an increase in sensitivity to ICI among such histologies as ASPS, UPS and DDLPS^[14,27,28]^. 

Given that T cell responses are central to the efficacy of ICI, there have been several studies evaluating tumor infiltrating lymphocytes (TILs) within sarcomas prior to and on treatment. D’Angelo^[29]^ and colleagues evaluated TILs in 50 sarcoma specimens to further evaluate the immune milieu. They noted infiltration of TILs and TAMs in 98% and 90% of tumors, respectively. They evaluated subsets of TILs with the median number of each subset and noted CD3+ cells 3.3% (0%-33.2%), CD8+cells 1.2% (0%-14%), CD4+ 0.2 (0%-13.6%), and FOXP3+ 0.1 (0-3.6%)^[29]^. There was an increased number of CD8+ cells in larger tumors or those who presented with metastatic disease, which is potentially indicative of T-cell exhaustion^[29]^. This study found that low CD3+ and CD4+ correlated with better survival, although contrary findings have been noted in other studies. In a cohort of 128 high-grade STS, increased density of CD8+ and CD3+ TIL infiltrates were associated with favorable OS, DSS and DFS when compared to low-density CD8+ and CD3+ infiltrates^[30]^. SARC028 correlative analysis evaluated changes in tumor-associated immune infiltrates from baseline to early on-treatment biopsies. They found that both effector memory cytotoxic T-cells and Tregs were subsequently increased after PD-1 blockade (*P *= 0.054)^[28]^. Analyses also showed that higher Treg percentages and higher density of cytotoxic T-cell infiltrates at baseline had longer median PFS^[28]^. A retrospective study of 81 patients with liposarcoma, LMS, UPS, and SS found that more highly mutated STS subtypes expressed higher levels of genes related to antigen expression and T-cell infiltration and inflammation^[5]^. Higher expression was seen in UPS and LMS compared to SS. They also had higher levels of *CD3D*, a marker for T cell infiltration, and *CD8A*, a marker for CD8+ T-cells^[5]^. 

PD-L1 expression in sarcoma is varied, and the data regarding the correlation between PD-L1 expression and responsiveness to ICI in STS is variable, and at this time there is not a consistent correlation^[5,29,30]^. In a cohort of resected UPS, SS, AS, ASPS, and ES, any level of PD-L1 expression in tumor cells was positive in 28.1% (*n *= 36/128) of cases. The highest level was found in UPS and the patients with UPS who were PD-L1 positive were noted to have better OS and PFS compared to those patients who were PD-L1 negative^[30]^. An analysis of 50 sarcoma specimens noted tumor cell PD-L1 expression of 12% and lymphocyte and macrophagic PD-L1 expression in 30% and 58% of specimens respectively with no correlation with prognostic indicators^[29]^. Pollack and colleagues evaluated PD-1 expression by immunohistochemistry (IHC) in common STS subtypes and scored them from 0 to ≥ 5. 35% (*n *= 28) of tumors had PD-L1 expression, and 51% (*n *= 41) had PD-1 expression of ≥ 2, with UPS associated with higher expression levels of both PD-L1 and PD-1. Although this study was not designed to evaluate survival outcomes, no correlation was found^[5]^. A pooled analysis of sarcoma clinical trials evaluated PD-L1 expression (≥ 1%) in soft tissue sarcoma. This was observed in 13.6% (*n *= 21/156) of patients with available data, and in this group, there was a corresponding ORR of 30% in PD-L1 positive tumors. In the PD-L1 negative tumors, the response rate was 7%^[31]^. Correlative analysis from the SARC028 trial noted PD-L1 positivity in 5% of tumors. Both of the PD-L1 positive tumors were UPS and responded to pembrolizumab, although five other patients responded and were PD-L1 negative^[28]^. Given the varied expression of PD-L1, particularly between different histologic subtypes, the prognostic and predictive significance of PD-L1 expression remains indeterminate^[12]^. 

The sarcoma TME varies widely by histology and can influence outcomes in patients with sarcoma^[23]^. Within the TME, immunosuppressive cytokines, abnormal perfusion from tumor angiogenic networks and metabolic conditions can inhibit T-cell infiltration and function. LMS have poor responses to ICI, and previous studies have shown poor T-cell infiltration in these tumors^[29,31]^. Gene expression profiling has also revealed high-level expression of macrophage-associated genes, such as CD 163 and CD68, which was associated with worse disease-specific survival in nongynecologic LMS^[32]^. Petitperez and colleagues studied TME gene expression profiles within STS based on immune classifications from immune low to immune high and highly vascularized^[33]^. They found most LMS classified to the low immune classes (classes A&B), DDLPS in the highly vascularized group (class C) and immune high (classes D&E) distributed across a variety of histologies^[33]^. The immune high group (E) is characterized by tertiary lymphoid structures rich in B-cells. Despite high or low CD8+ cell density, the presence of B-cells was the strongest prognostic factor with improved survival and high response to pembrolizumab therapy. 

In those patients who initially respond to ICI, a proportion will eventually progress after ICI. Many of the mechanisms are similar to de novo resistance, but much remains unknown regarding the mechanism behind acquired resistance within sarcoma. There are several potential mechanisms including downregulation of tumor antigen presentation and subsequent lack of T-cell recognition, loss of T-cell function and development of escape mutation variants^[25]^. The immunoediting hypothesis refers to the interactions between the immune system and tumor cells that eventually lead to the inability of the immune system to recognize the tumor^[34,35]^. Anagnostou and colleagues matched pre-treatment and ICI resistant non-small cell lung cancer and found that resistant tumors had a loss of 7-18 putative neoantigens, many of which generated peptides responsible for host immune response^[36]^. There have been studies in melanoma regarding acquired resistance to PD-1 blockade. Zaretsky and colleagues performed whole-exome sequencing on the paired baseline and relapsing biopsy samples in four patients with melanoma who had initially responded to pembrolizumab therapy^[37]^. Two of the four patients revealed loss-of-function mutations in genes encoding interferon-receptor-associated Janus Kinase 1 (JAK1) or JAK2. This resulted in insensitivity to the antiproliferative effect of interferon on cancer cells. They also noted mutations in beta-2-microglobulin which led to the loss of major histocompatibility complex class 1 surface expression^[37]^. 

## BIOMARKERS OF RESPONSE

A focus of sarcoma research has been on predictive biomarkers which may delineate those who are likely to respond to ICI [Table 2], although predictive biomarkers remain elusive, and to date, there are no clearly defined biomarkers for soft tissue sarcoma. 

**Table 2:** ICI biomarkers in soft tissue sarcoma

**PD-1/pd-l1 expression**
cd8+ T-cells
Regulatory T cells
Tumor-associated macrophages
Tumor mutational burden
Neutrophil-to-lymphocyte ratio
DNA methylation profiles
Sarcoma Immune Class

Prior studies have noted that a high baseline neutrophil-to-lymphocyte ratio was associated with aworse prognosis in sarcoma^[27,38,39]^. The increased neutrophil-to-lymphocyte ratio has also been associated with inferior PFS in sarcoma patients who were treated with axitinib and pembrolizumab, but further elucidation of whether this is specifically predictive in the setting of ICI^[27]^. Sarcoma patients who were treated with axitinib and pembrolizumab were also noted to have improved outcomes if they had higher plasma angiogenic activity at baseline^[27]^. There is still further investigation to elucidate the prognostic implications of this finding.

There is interest in the emergence of DNA methylation profiles as predictive biomarkers in sarcoma patients, particularly those treated with ICI. DNA methylation has been implicated in tumorigenesis in a variety of tumors including sarcoma. A recent retrospective analysis of 35 recurrent sarcoma patients who were treated with anti-PD-1 ICI, most of which were treated with Pembrolizumab, noted DNA methylation differences between responders and nonresponders^[23]^. The most prominent pathway differences were seen in Rap 1 signaling, focal adhesion, adherens junction, pathways in cancer and extracellular matrix -receptor interaction^[23]^. In this study, PD-L1 expression and density of TIL subsets were evaluated and there was no correlation with response to ICI^[23]^. DNA methylation profiling was evaluated in 36 angiosarcoma specimens and revealed two subtypes (A and B) which were divided into four subclusters. Survival analysis showed better overall survival in cluster A at 22 months compared to cluster B at 6 months (*P *= 0.046)^[40]^. 

In other cancer types, there have been established biomarkers that predict response including tumor mutational burden and expression of the immune checkpoint molecules PD-1/PD-L1^[26]^. 

A retrospective study by Lu and colleagues of 18 metastatic sarcoma patients receiving anti-PD 1 therapy low TMB in all patients (range 1.12-3.45 mutations/MBs)^[41]^. Within sarcoma, PD-L1 positivity rates are low and have not been noted to be a consistent biomarker^[28,29]^. Data has been inconsistent with some studies noting improved survival in PD-L1 positive patients^[31]^ and several without correlation between PD-L1 expression and outcomes^[5,28]^. A retrospective study of 18 metastatic sarcoma patients receiving anti-PD 1 therapy noted a PR in 22.2% (4/18) and SD in 50% (9/18) at 12 weeks with an ORR of 18.3% in soft tissue sarcomas. Whole exome sequencing was performed pre-treatment in 8 patients and did not note associations of PD-L1 expression with clinical response^[41]^. These studies highlight the unreliability of PD-L1 as a biomarker in STS. 

Rates of TILs and TAMs have also been evaluated as potential biomarkers for response. Correlative analysis of SARC028 noted a higher percentage of tumor immune cell phenotypes in those patients who had responses to pembrolizumab^[28]^.

Immunohistochemical staining can be used to confirm CD68 and CD163 positive macrophages. As noted above, a subset of non-gynecologic LMS has been noted to have dense infiltrates of these TAMs were found to have shorter disease-specific survival, although this was not seen in uterine LMS^[32]^. LMS has been noted to have high levels of T-cell-related gene expression, and it is postulated that TAMs are likely critical to immune invasion in these tumors given the poor clinical outcomes with single-agent ICI in LMS^[5,21,32]^. 

The TME has also recently been of interest as a prognostic indicator of response to ICI. STS biopsies from the SARC028 clinical trial were placed into their sarcoma immune classes and the ORR in group E was 50% (*n *= 5/10), followed by group D of 25% (*n *= 3/12) and group C of 22% (2/9)^[33]^. These are all higher than the ORR of 21.2% in the overall cohort. There were no responders in groups A and B. Group E also had improved PFS when compared to groups A &B (*P* = 0.023 and *P* = 0.0069, respectively)^[33]^. This study has laid the groundwork for potentially risk stratifying patients prior to treatment and identifying those who would be more likely to respond. 

## OVERCOMING RESISTANCE

**Figure 1:**
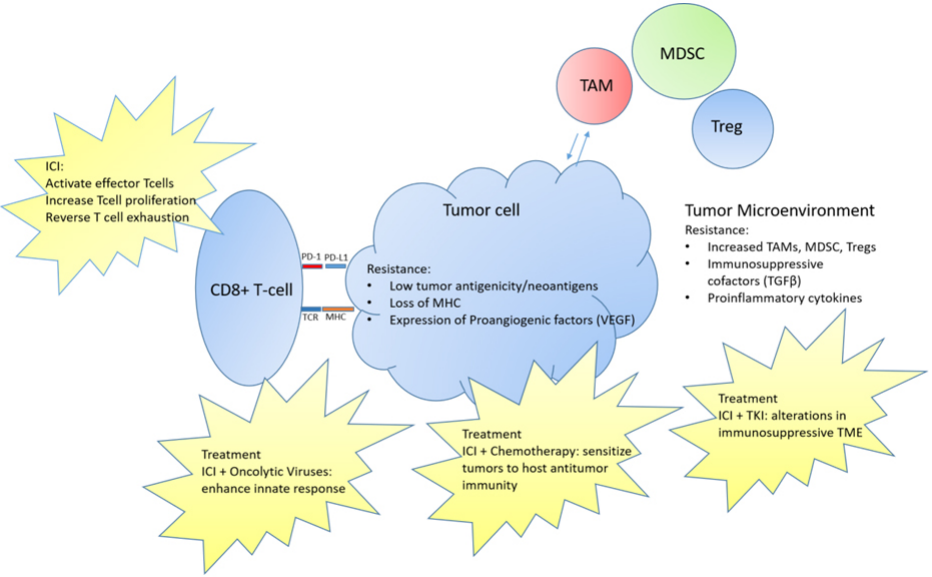
Resistance mechanisms to immunotherapy in soft tissue sarcoma with current treatment mechanisms aimed at overcoming resistance. ICI: Immune checkpoint inhibitor; MDSCs: myeloid-derived suppressor cells; MHC: major histocompatibility complex; PD-1: programmed cell death 1; TAMs: tumor-associated macrophages; TCR: T cell receptor; TKI: tyrosine kinase inhibitor; TME: tumor MIcroenvironment.

Given the relatively low response rates to ICI in soft tissue sarcomas, there has been interest in manipulating the immune environment to increase responses [Figure 1]. Many sarcomas have limited neoantigens and therefore, limited immunogenicity without the generation of tumor-specific T-cells. There are several approaches that are combined with ICI to overcome this limitation including cytotoxic chemotherapy and oncolytic viruses. There is also interest in using drugs, such as tyrosine kinase inhibitors (TKIs) in combination with ICI to target the TME and overcome its suppressive influences which is mediated through immunosuppressive immune cells and cytokines. 

Cytotoxic chemotherapy is an effective inducer of immunogenicity and increases inflammatory cytokines. There is evidence, using lung adenocarcinoma models, that chemotherapy can sensitize tumors to host antitumor T-cell immunity^[42]^. A lung adenocarcinoma mouse model was able to show that an antitumor CD8(+) T-cell response could be induced with immunogenic chemotherapy^[42]^. Within sarcoma, there have been several trials combining chemotherapy with immunotherapy to this end. The SAINT trial evaluated the combination of trabectedin (1.2 mg/m^2^ IV q 3 weeks) with nivolumab (3 mg/mg q 2 weeks) and Ipilimumab (1 mg/m^2^ q 12 weeks)^[19]^. Forty-one patients with previously untreated advanced or metastatic soft tissue sarcoma were included. The overall response rate was 19.5% with a disease control rate of 87.8%. Median PFS was > 6*.*0 months and median OS was > 12.5 months^[19]^. These data suggest synergy with trabectedin and Ipilimumab and Nivolumab. A phase 1/2 study by SM Pollack and colleagues evaluated the combination of doxorubicin (45 and 75 mg/m^2^ q3 weeks) and pembrolizumab (200 mg q3 weeks) in 37 patients with advanced sarcoma who had not received prior anthracycline^[17]^. The ORR was 13% for phase 2 patients and 19% overall with a median PFS of 8.1 months (95%CI: 7.6-10.8) and median OS 27.6 mo (95%CI: 18.7-not reached). While this study did not reach its primary endpoint (ORR of 15% with 85% power), there was a clinically significant increase in PFS compared to historical studies. Two of three patients with UPS and two of four patients with dedifferentiated LPS had durable partial responses. In 29 patients, there was evaluate IHC for correlatives. 66% had a PD-L1 score of 0, and PD-L1 was not associated with PFS or OS. Tumor-infiltrating lymphocytes were present in 21% of evaluable tumors and associated with inferior PFS (*P* = 0.03). They assessed serum cytokine levels before treatment and during the first two cycles. Granulocyte macrophage colony-stimulating factor levels increased each cycle, and IL-15 levels dropped following doxorubicin treatment. Circulating IL-2R, IP10, and CD30 levels rose sharply after cycle one and levels of IL-8 dropped^[17]^.

Oncolytic viruses, engineered viral vectors that selectively infect and replicate within cancer cells, are also being combined with checkpoint inhibitors in the treatment of sarcomas^[15]^. The innate immune system is also able to recognize these viruses as foreign and initiate an immune response^[43]^. A recent phase II study evaluated the combination of intralesional talimogene laherparepvec (T-VEC, the first dose, ≤ 4 mL × 10^6 ^plaque-forming units [PFU]/mL; second and subsequent doses, ≤ 4 mL × 10^8^PFU/mL injected into palpable tumor site(s) on day 1 of each 21-day cycle) with pembrolizumab (200 mg/dose q 3 weeks) in 20 patients with locally advanced or metastatic sarcoma^[18]^. The best ORR at 24 weeks was 30 % (*n *= 6, 95%CI: 12%-54%) and overall was 35% (*n* = 7, 95%CI: 15%-59%). Median PFS was 17.1 weeks (95%CI: 12.6-NR weeks) and median disease-specific survival was 74.7 weeks (3-sided 95%CI: 49.0-NR weeks). Two of the patients who responded to treatment had disease progression while on ICI prior to entering the study, which may suggest synergism between ICI and T-VEC. There were 11 patients with paired evaluable tumor samples and 55% (*n *= 6) converted from PD-L1 negative at baseline to PD-L1 positive after treatment. Six of the seven patients who responded had evaluable tissue, and in this cohort, there were one PD-L1 positive baseline tumor and four PD-L1 positive post-treatment tumors^[18]^. No patient tumors in the refractory group (*n *= 13) were PD-L1 positive at baseline and five were positive after treatment. They also evaluated tumor-infiltrating lymphocyte (TIL) scores which were higher in the response groups (mean TIL score 3) compared to the unresponsive group (mean TIL score 2)^[18]^. Responsive patients also had the presence of aggregates of CD3+/CD8+ TILs in the tumor on the pre-treatment biopsy, particularly at the infiltrating edge, and this number increased in the post-therapy samples. Comparatively, there were minimal CD3+/CD8+ infiltrates in the nonresponsive patient tumors. This is a potentially promising therapy and additional investigation is ongoing in sarcoma.

There have been several studies evaluating TKIs in combination with ICI to overcome the immunosuppressive microenvironment. There are several well-known mediators of this environment including vascular endothelial growth factor (VEGF) and Transforming growth factor-ß. VEGF and other proangiogenic cofactors are necessary for tumor growth and spread. TKIs with activity against these factors have produced responses in metastatic sarcoma in prior studies^[44-46]^. An in vitro study of cocultures of sarcoma evaluated the role of TKIs and PD-1 based therapy^[47]^. In this study, human osteosarcoma and SS cell lines were treated with sunitinib. They were then cocultured with dendritic cells (DCs) and the phenotype of these DCs was determined by flow cytometry. Mature DCs were cultured with autologous T cells and the T cells were evaluated for PD-1 expression, proliferation, Treg induction, and IFN-γ production, before and after nivolumab exposure. They found that treatment with sunitinib induced upregulation of PD-L1 on sarcoma cells, induced maturation of DCs, and reduced Treg induction. There was no effect on T cell proliferation or T cell subpopulations. Treatment with nivolumab induced IFN-γ-producing effector T cells^[47]^. 

A phase II single-arm study by Wilky *et al.* combined axitinib (5mg twice daily), an oral TKI, with pembrolizumab (200mg/dose on day 8 and every 3 weeks for up to 2 years) in 33 patients with advanced sarcoma^[27]^. ORR was 25% (*n *= 8, 95%CI: 12.1-43.8) with clinical benefit rate of 53.1% (*n *= 17; 95%CI: 35.0-70.5). In the intention to treat analysis, median PFS was 4.7 months (95%CI: 3.0 to 9.4) and median OS was 18.7 months (95%CI: 12.0 to NR). In this study, 11 patients had alveolar soft part sarcoma (ASPS). ASPS is a rare translocation-driven sarcoma subtype that frequently presents in adolescents and young adults. There is a growing body of evidence that these tumors are responsive to both TKIs and ICI and several studies are ongoing^[48]^. In this study, the ORR in the ASPS cohort was 54.5% (95%CI: 24.6-81.9). The response rate in the ASPS was greater than that would be expected with either axitinib or pembrolizumab alone, and four of five patients who achieved a partial response had not achieved a partial response with at least on previous TKI. Correlatives and exploratory analyses are still underway.

A recent phase Ib/II trial evaluated the combination of nivolumab (3 mg/kg IV on day 15, then every 2 weeks) with sunitinib (37.5mg for the first 14 days, then 25mg per day) in 68 patients with advanced soft tissue sarcoma who had progressed on prior therapy^[16]^. The 6-month PFS was 48% (95%CI: 41-55%) with a median PFS of 5.6 months (3.0-8.1). The median overall survival was 24 months with an 18-month survival of 67% (95%CI: 59-74%). The ORR was 21%, with 100% of responding patients alive at 18 months. These response rates, PFS and OS are favorable compared with activity in anti-PD-1 or sunitinib monotherapy in previous trials^[13,49]^. 

These combination trials show promise in the quest to overcome resistance innate to many sarcomas. Further combination trials are underway. 

## CONCLUSION

Treatment of sarcomas remains difficult given the heterogeneity in immunogenic features of histologic subtypes and varied responses to ICI due to underlying primary or acquired resistance. There remains interest and promise in combining ICI and immunosensitizing agents to overcome underlying resistance mechanisms within sarcomas and the TME. Further, identifying reliable biomarkers to determine who responders to ICI will be remains an important but complex undertaking. Ongoing studies to better define the immunologic landscape, the immunosuppressive role of the TME and subsequent resistance mechanisms will improve understanding of this complex disease with the goal of improving clinical outcomes. 

## DECLARATIONS

### Copyright

© The Author(s) 2022.
